# Dual quantum cascade lasers for noninvasive glucose detection using photoacoustic spectroscopy

**DOI:** 10.1038/s41598-023-34912-3

**Published:** 2023-05-16

**Authors:** Abdulrahman Aloraynan, Shazzad Rassel, Md. Rejvi Kaysir, Dayan Ban

**Affiliations:** 1 Department of Electrical Engineering, Umm Al-Qura University, Makkah, Saudi Arabia; 2 Department of Electrical and Computer Engineering, University of Waterloo, Waterloo, ON N2L 3G1 Canada; 3 Waterloo Institute for Nanotechnology, University of Waterloo, Waterloo, ON N2L 3G1 Canada

**Keywords:** Biomedical engineering, Electrical and electronic engineering, Electronics, photonics and device physics, Optical physics, Health care, Optics and photonics

## Abstract

The combination of mid-infrared and photoacoustic spectroscopy has shown promising developments as a substitute for invasive glucose detection technology. A dual single-wavelength quantum cascade laser system has been developed using photoacoustic spectroscopy for noninvasive glucose monitoring. Biomedical skin phantoms with similar properties to human skin have been prepared with blood components at different glucose concentrations as test models for the setup. The detection sensitivity of the system has been improved to ± 12.5 mg/dL in the hyperglycemia blood glucose ranges. An ensemble machine learning classifier has been developed to predict the glucose level in the presence of blood components. The model, which was trained with 72,360 unprocessed datasets, achieved a 96.7% prediction accuracy with 100% of the predicted data located in zones A and B of Clarke’s error grid analysis. These findings fulfill both the US Food and Drug Administration and Health Canada requirements for glucose monitors.

## Introduction

Researchers have explored various techniques for noninvasive glucose detection, including electromagnetic sensing^1,2^, impedance spectroscopy^3,4^, electrochemical sensing^5,6^, and Raman spectroscopy^7,8^. Nevertheless, none of these approaches have met the physiological necessity requirements because of their low accuracy or operational instability^9^. Other minimally invasive approaches have been developed; however, they demand iterative surgical implantation for the sensors, which raises the skin irritation dilemma^10^. Infrared (IR) spectroscopy, including the MIR and NIR regions, is being developed as a promising alternative technique to invasive glucometers^11,12^. Both NIR and MIR regimes demonstrate broad and strong glucose fingerprint absorption. Moreover, the MIR region has specific glucose fingerprints with narrower interference with other blood components compared to the NIR region^13,14^.

The combination of MIR and photoacoustic (PA) spectroscopy has shown promising developments in recent years as a substitute for invasive glucose monitoring technology^15–18^. PA spectroscopy utilizes the vibration modes of the glucose molecules in the MIR region as an alternative approach to compensate for the optical losses in the transmission and absorbance spectroscopy. Quantum cascade lasers (QCLs) in the MIR region have the advantage of generating strong and stable PA signals. The acoustic signals generated by QCLs can reach the interstitial fluid (ISF) of the human skin, where the glucose is diffused in the epidermis layer^19^. These acoustic signals are eventually collected by a sensitive microphone to show a direct relationship to the blood glucose level.

The combination of MIR and PA spectroscopy for noninvasive glucose detection was first explored by Lilienfeld-Toal et al. in 2005^15^. Two single-wavelength QCLs were used, one at the glucose absorption peak at 1080 cm$^{-1}$ and the second one as a reference at 1066 cm$^{-1}$. A correlation factor ($R^2$) of 0.61 was achieved for in vivo measurements. In 2011, Pleitez et al.^17^ employed three QCLs to detect the glucose concentration in the palm at two glucose peaks (1084 and 1054 cm$^{-1}$) and 1100 cm$^{-1}$ for the background. A twin Helmholtz gas cell was used, and the correlation factor was enhanced to 0.7. In vitro measurements were conducted by Kottmann et al.^16^ using a broadly tunable external cavity (EC) QCL. A glucose detection limit of ± 100 mg/dL was acquired with a correlation factor of 0.998.

In 2013, Kottmann et al.^20^ used silver halide optical fiber for light transmission in order to improve the detection sensitivity to ± 57 mg/dL with $R^2$ = 0.993 in an aqueous glucose solution. Three years later, the same research group employed a dual-wavelength technique at 1080 and 1180 cm$^{-1}$ for in vivo measurements^19^. The prediction limit was raised to ± 30 mg/dL for a glucose level between 90 and 170 mg/dL at a 90% confidence level. Recently, the detection sensitivity was enhanced to ± 25 mg/dL using a single wavelength QCL at 1080 cm$^{-1}$ in artificial skin phantoms with the employment of machine learning^18,21^. Nevertheless, for clinically approved glucometers, the detection sensitivity has to be ± 15 mg/dL, according to the US Food and Drug Administration (FDA) and Health Canada^22,23^. Table 1 summarizes recent progress in the PA and MIR spectroscopy for glucose detection.

**Table 1:** Recent progress in PA and MIR combined spectroscopy for glucose detection.

Date	Reference	Light source	Wavenumber ($\hbox {cm}^{-1}$)	Measurements	Glucose (mg/dL)	Correlation or sensitivity	ML.	Main contributions
2005	Toal et al.^15^	QCL	P:1080 Bg:1066	Forearm	0–300	R = 0.61	No	Combination of PA & MIR
2012	Kottmann et al.^16^	QCL	P:1034	Epidermal samples	0–2000	$\pm \,100$ mg/dL	No	Use of tunable QCLs & N$_2$ ventilation
2012	Pleitez et al.^17^	EC-QCL	P:1054 &1084 Bg:1100	Palm	80–260	R = 0.70	Reg.	Selection of three wavelengths
2013	Kottmann et al.^20^	EC-QCL	P:1034	Glucose solution	0–5000	$\pm \,57$ mg/dL	No	Use of fiber optics for light delivering
2013	Pleitez et al.^24^	EC-QCL	1000–1220	Hypothenar	40–240	–	Reg.	Noise removal by multivariate models
2016	Kottmann et al.^19^	EC-QCL	P:1080 Bg:1180	Forearm and fingertip	90–170	± 30 mg/dL	Reg.	Improved stability by increasing pulse rate
2018	Sim et al.^25^	EC-QCL	950–1245	Palm and fingertip	100–250	70% in zone A	Reg.	Study of skin effect on measurement
2022	Aloraynan et al.^18^	QCL	P:1080	Skin phantoms	75–300	± 25 mg/dL	Yes	Enhanced detection sensitivity

*ML.* machine learning, *P* peak, *Bg* background, *Reg.* regression only

In this paper, the glucose detection sensitivity of the MIR and PA spectroscopy has improved to ± 12.5 mg/dL using dual single-wavelength QCLs (1080 and 970 cm$^{-1}$) for noninvasive glucose monitoring. Artificial skin phantoms with similar properties to human skin have been prepared with blood components at different glucose concentrations from 100 to 275 mg/dL as test models for the system. This glucose range covers the scope of interest for blood glucose levels in healthy individuals and those living with diabetes. The dual QCLs system demonstrates sustainability in detecting glucose concentrations in the presence of albumin, sodium lactate, cholesterol, and urea. A machine learning (ML) classifier model has been developed to predict the glucose level of skin samples. The model has achieved 96.7% prediction accuracy for samples with and without blood components, with 100% of the predicted data located in zones A and B of Clarke’s error grid analysis (EGA).

## Results and discussion

**Figure 1:**
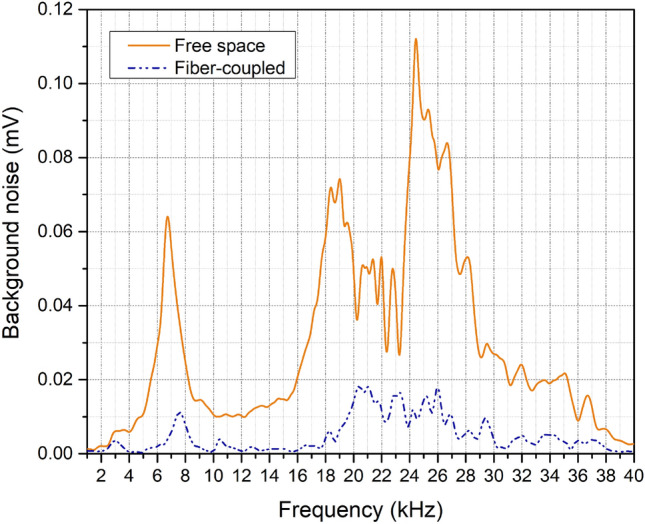
Background noise in the free space and fiber-coupled light transmission.

The transformation from free space to fiber optics coupling for light transmissions enhanced the SNR of the system with an average of 81% for the entire spectrum, as shown in Fig. 1. The suspension of the background noises aids in quantifying the glucose signals in the investigated acoustic range. Such an enhancement facilitated achieving the objective of improving the detection sensitivity of the setup to ± 12.5 mg/dL. Furthermore, it assisted in refining the correlation factor between the detected glucose signals.

**Figure 2:**
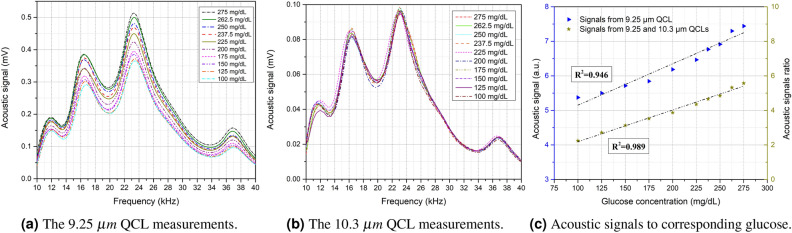
Measurements of the first set of glucose phantoms from 100 to 275 mg/dL.

Figure 2a, b show the acoustic signals obtained from the first set of glucose phantoms for the 9.25 $\upmu$m and 10.3 $\upmu$m QCLs, respectively. The figures show the average of the 10 rounds of the two days’ measurement for each light source per glucose concentration. The glucose difference in the samples was set to ± 25 mg/dL in the non-diabetic range and ± 12.5 mg/dL in the diabetic range, aiming to achieve a detection sensitivity that fulfills the FDA and Health Canada requirements^22,23^. For the 9.25 $\upmu$m QCL, leasing at the glucose fingerprint, phantoms with higher glucose concentrations are expected to generate stronger acoustic signals due to the higher absorption of light by the glucose molecules. The third peak of the collected acoustic spectrum, ranging from 21 to 25 khz, was found to be the most closely correlated to the glucose differences in the samples. In contrast, signals from the 10.3 $\upmu$m QCL, as intended, have not shown a positive correlation to the glucose differences in the phantoms. This is because the 10.3 $\upmu$m wavelength does not respond to the glucose molecules. Hence, this wavelength can be used as a reference to subtract the background effects.

After obtaining the acoustic spectrum of the glucose samples for both QCLs, the area under the curves was integrated to show the relationship between the acoustic signal to the corresponding glucose phantoms. Figure 2c shows two cases of the results, one when using only the 9.25 $\upmu$m QCL and the other when using two QCLs. As for the first case, the integrated signals from the 9.25 $\upmu$m laser show a positive correlation between the acoustic signals and the glucose concentration with a linear correlation factor of 0.946. The 9.25 $\upmu$m light source was able to detect the glucose differences in the samples with ± 25 and ± 12.5 mg/dL glucose differences. The average resolutions between the acoustic signals of two glucose samples with ± 25 and ± 12.5 mg/dL are 3.8% and 3.5%. These resolutions indicate inconsistency in the differences of the glucose acoustic signals, which shows the importance of adding a reference QCL that does not correspond to glucose molecules to enhance the consistency of the differences. As a result, the 10.3 $\upmu$m QCL was added to the system as a reference light source. The results of two QCL measurements improved the average resolutions of the glucose samples with ± 25 and ± 12.5 mg/dL to 4.8% and 2.7%, which enhanced the correlation factor to 0.989, as shown in the second case of Fig. 2c. Moreover, the standard deviation of the results was reduced from 1.51 using a single QCL to 0.79 using two QCLs. The standard deviation was assessed to determine the fluctuation in the differences between the glucose samples for the entire set. In this experiment, the detection sensitivity was improved to ± 12.5 mg/dL for the unprocessed data using a single wavelength QCL. The detection sensitivity was further enhanced by adding a reference wavelength to the system to subtract the background effect.

**Figure 3:**
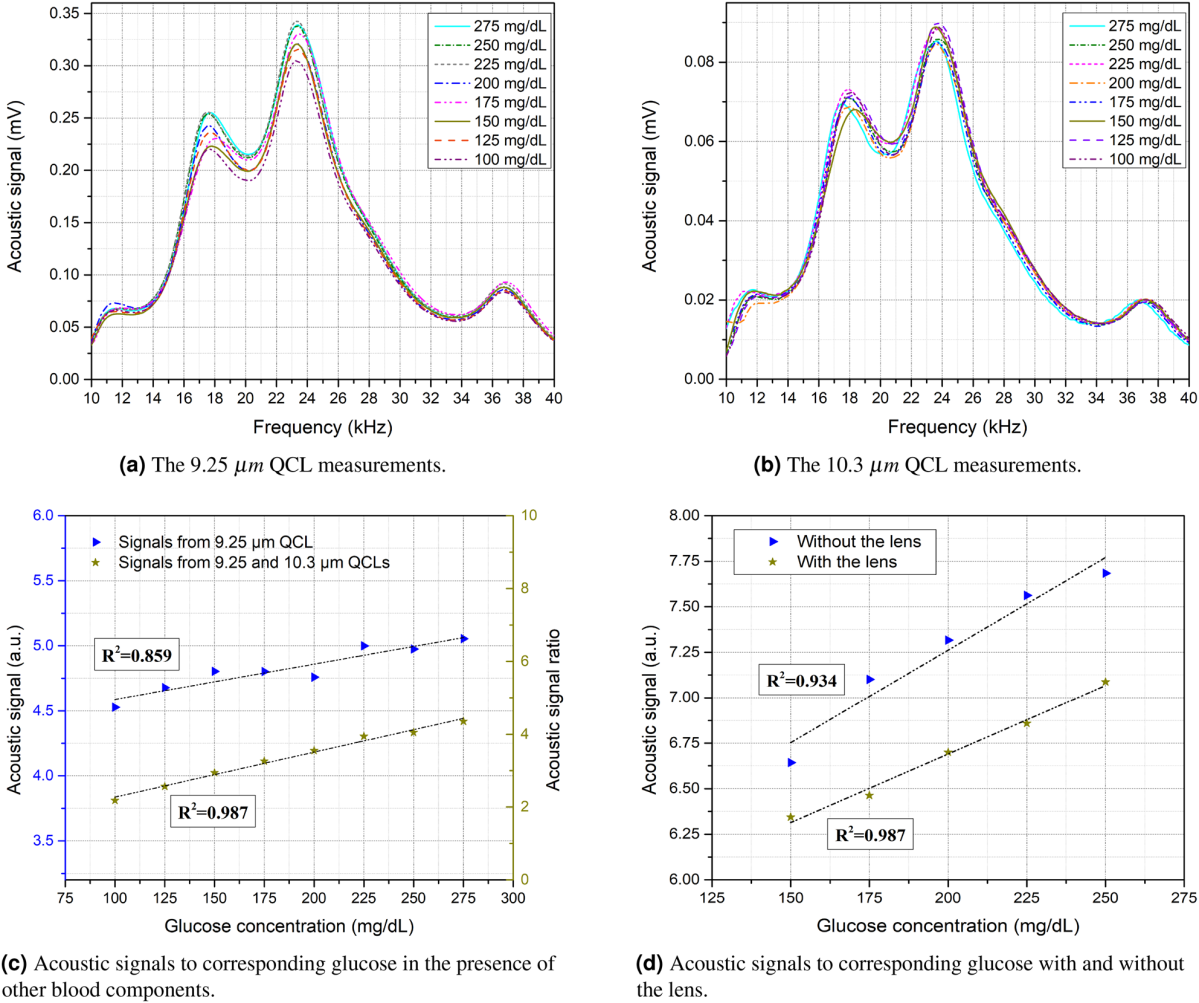
Measurements of the second set of glucose phantoms with blood components from 100 to 275 mg/dL.

The second set of glucose phantoms was prepared by adding other blood components, namely albumin, sodium lactate, cholesterol, and urea. These blood components were added simultaneously to the glucose samples ranging from 100 to 275 mg/dL. The aim was to examine the detection capacity of the system in the presence of these components that may interfere with the glucose in the in vivo measurements. Figure 3a, b show the average of 10 rounds of acoustic signals per glucose concentration of the two days’ measurement using the 9.25 $\upmu$m and 10.3 $\upmu$m QCLs. The results show that the blood components that were added to the samples interfered with the glucose signals, which lowered the detection efficiency of the 9.25 $\upmu$m QCL. The impact of the blood components distinctly reveals in the linear correlation factor when the area under the curve of the acoustic spectrum is integrated. Figure 3c shows the relationship between the acoustic signals to the corresponding glucose phantoms using single and dual QCLs. When only the 9.25 $\upmu$m QCL was used, the system failed to detect some of the glucose samples correctly. The linear correlation factor of using only the 9.25 $\upmu$m QCL is 0.859 with 2.01% average resolution between the glucose samples. When the 10.3 $\upmu$m QCL was used as a reference in the measurements, the correlation factor enhanced to 0.987 with a 2.15% average resolution. The standard deviation of the measurements was reduced from 1.79 using a single QCL to 0.72 using two QCLs. Therefore, in this case, adding a reference light source is essential for eliminating the signals that are received from blood components other than glucose. Moreover, the reference light source assists in subtracting the effects of environmental conditions that vary over the experiment, such as temperature and humidity, to maintain the results compatible.

The lens effect on the accuracy of the measurements has been studied in order to determine the necessity of installing an optical lens into the system. The primary objective of the lens is to allow both QCLs to radiate at the exact spot of the samples, which assists the detection selectivity in the case of non-uniform distribution of the blood components. Figure 3d shows the relationship between the acoustic signals to the corresponding glucose samples using dual QCLs with and without an optical lens. The system was able to detect glucose in the presence of the blood components in both cases. Nevertheless, installing an optical lens enhanced the standard deviation in the consistency of the differences by 60%. Furthermore, the correlation factor was improved from 0.934 to 0.987 when the lens was installed into the system. Table 2 summarizes the results of the two glucose phantoms and the lens effect.

**Table 2:** Summary of the results.

Measurements objective	Glucose level (mg/dL)	Sample contents	QCL(s)	Correlation factor ($R^2$)	Differences standard deviation ($\sigma$)
± 25 and ± 12.5 mg/dL detection sensitivity using a single wavelength	100:25:225 and	Glucose only	9.25 $\upmu$m	0.946	1.51
	225:12.5:275				
± 25 and ± 12.5 mg/dL detection sensitivity using two wavelengths	100:25:225 and	Glucose only	9.25 & 10.3 $\upmu$m	0.989	0.79
	225:12.5:275				
Glucose detection in the presence of blood components	100:25:275	Glucose, albumin, urea,	9.25 $\upmu$m	0.859	1.79
		Sodium lactate, and cholesterol			
Glucose detection in the presence of blood components	100:25:275	Glucose, albumin, urea,	9.25 & 10.3 $\upmu$m	0.987	0.72
		Sodium lactate, and cholesterol			
Lens effect on the detection selectivity	150:25:250	Glucose, albumin, urea,	9.25 & 10.3 $\upmu$m no lens	0.934	2.24
		sodium lactate, & cholesterol	9.25 & 10.3 $\upmu$m with lens	0.987	0.83

### Machine learning classifier model

**Figure 4:**
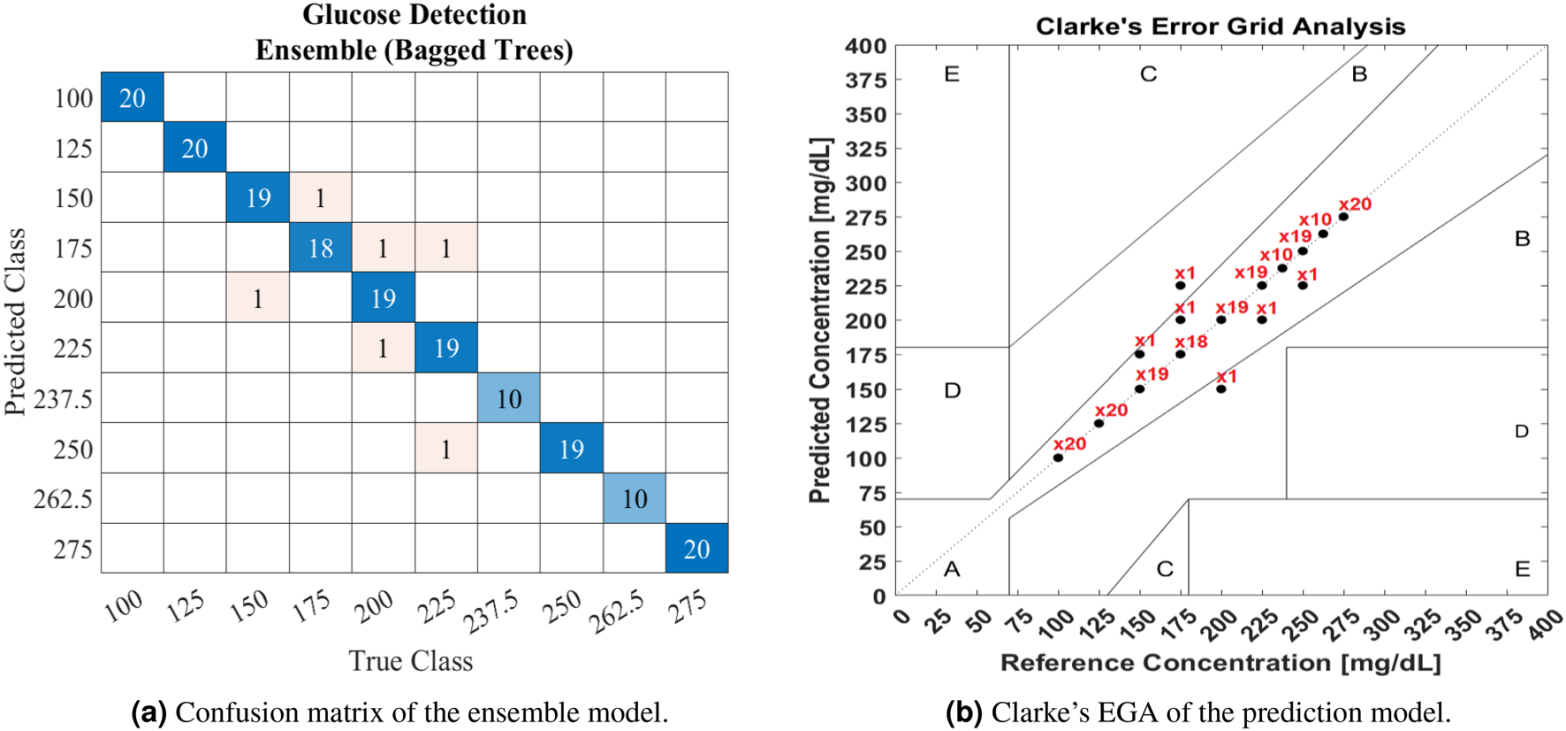
Performance of the developed ensemble classification model.

After establishing the system’s feasibility in detecting glucose in the skin phantoms with and without blood components using dual QCLs, an ensemble classification model was developed using the unprocessed data. The model was trained with the raw acoustic signals of both QCLs with 72,360 datasets. The ensemble classifier achieved a prediction accuracy of 96.7% using a decision tree of 30 learners and a maximum split number of 180. The confusion matrix of the ensemble classifier is shown in Fig. 4a. The confusion matrix visualizes the classifier’s performance by representing the predicted classes versus the reference classes. The confusion matrix was then converted into Clarke’s EGA, as shown in Fig. 4b, to evaluate the model according to the FDA requirements. The figure represents the number of times the classifier predicts the glucose class for each data sample. This results in 98.89% of the predicted results being located in zone A, while 1.11% is in zone B. Thus, the developed model qualifies for the FDA standards for glucose monitors, which require 99% of the predicted data to be in zones A and B. Further evolution in the ML model development is necessary for the in vivo measurements to build a robust model. For example, integrating feature selection algorithms can enhance the prediction accuracy of ML models and avoid over-fitting issues. Besides, pre-processing the data can assist in removing the noises generated from the background.

## Conclusion

A dual QCL system using photoacoustic spectroscopy was developed for noninvasive glucose detection. The developed system shows sustainability in detecting glucose for the skin phantoms using single and dual single-wavelength QCLs. The detection sensitivity of the system was enhanced to ± 12.5 mg/dL using a QCL lasing at a glucose fingerprint of 9.25 $\upmu$m with a correlation factor of 0.946. This correlation factor was improved to 0.989 when a reference QCL, lasing at 10.3 $\upmu$m, was added to the system. Furthermore, the dual QCL setup demonstrates promising results in detecting glucose in the presence of other blood components that interfere with the glucose fingerprint, namely, albumin, sodium lactate, cholesterol, and urea. A correlation factor of 0.987 was achieved for the two days’ measurements with a standard deviation of 0.72. An optical lens was integrated into the system to allow both lasers to radiate at the exact spot of the samples when the blood components were added to the samples. The correlation factor was enhanced from 0.934 to 0.987 when an optical lens was installed.

The 72,360 datasets obtained from the measurements were used to train an ensemble classification model. The acoustic signals from the two sets of phantom skin were used in training. The model successfully predicted the correct classes of phantoms with 96.7% accuracy. All predicted results were located in zones A and B in Clarke’s EGA. These findings satisfied both FDA conditions and were considered the final stage before conducting the in vivo measurements.

## Methods

**Figure 5:**
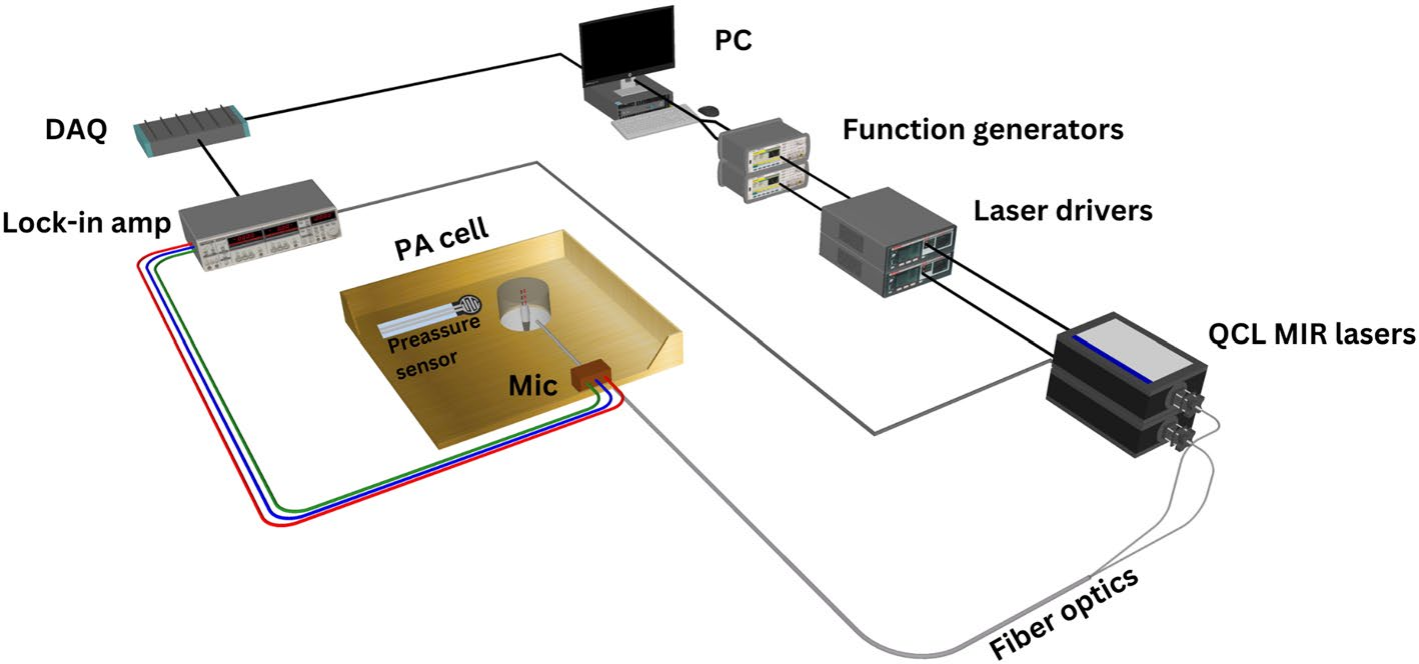
Schematic of the setup used for glucose detection using MIR and PA spectroscopy.

The experimental setup of the dual QCLs and PA spectroscopy for noninvasive glucose detection is shown in Fig. 5. In the developed setup, two single-wavelength QCLs were employed. The first QCL was at a glucose fingerprint of 9.25 $\upmu$m (1080 cm$^{-1}$) (QD9500CM1, Thorlabs, Newton, NJ, USA), and the second was employed as a reference to the measurements lasing at 10.3 $\upmu$m (970 cm$^{-1}$) (QD10500CM1, Thorlabs, Newton, NJ, USA). The threshold currents of the 9.25 $\upmu$m and 10.3 $\upmu$m QCLs are around 180 mA and 280 mA, respectively. The light beams of the dual QCLs were delivered to the PA cell using fiber optics cables (2FB-HF300LW-SMA-Bk0.5/0.5m, Guiding Photonics, United States). Both lasers were operated in pulsed mode at room temperature with a 40% duty cycle. The laser currents were frequency-modulated from 10 to 40 khz by a function generator (Agilent 55321A) with a 150 Hz frequency step.

Two main sets of artificial skin phantoms were prepared at different glucose concentrations to be used as the test models for the developed system. Both sets were prepared, ranging from 100 to 275 mg/dL in order to cover the range of interest for blood glucose levels in healthy individuals and those with diabetes. The glucose skin phantoms were prepared following the work of Lazebnik et al.^26^ and Aloraynan et al.^18^. The purpose of the first set was to examine the system’s sustainability in detecting glucose at ± 25 and ± 12.5 mg/dL glucose differences using single and dual QCLs. The first set was split into two categories: the non-diabetic range (100–225 mg/dL) and the diabetic range (225–275 mg/dL). The glucose difference was set to ± 25 mg/dL in the non-diabetic range, while in the diabetic range, it was reduced to ± 12.5 mg/dL. The differences were reduced in the diabetic range, aiming to achieve detection sensitivity within FDA specifications.

The second set of glucose phantoms was prepared by dissolving the blood components that interfere with the glucose fingerprint in the MIR region^27^, namely albumin, sodium lactate, cholesterol, and urea. The MIR spectra of glucose and these blood components are provided in^13^. The components were ground and soaked overnight in deionized water in order to create a uniform solution before adding them to the solution that produced the glucose phantoms. All components were added based on their normal range in human blood in order to examine the detection selectivity and sensitivity of the system using single and dual QCLs. Albumin occupies the largest share of the blood components’ weight at 4500 mg/dL, then cholesterol at 160 mg/dL, urea at 15 mg/dL, and sodium lactate at 12 mg/dL. After preparing a homogeneous solution, glucose was added to produce eight different concentrations from 100 to 275 mg/dL with a ± 25 mg/dL distinction. All blood components, including glucose, used in the sample preparation were purchased (Sigma-Aldrich, Canada). More information on the preparation of the artificial skin phantoms is provided in^18^.

### Glucose measurements

The first set of the prepared glucose phantoms, ranging from 100 to 225 mg/dL at ± 25 mg/dL and 225 to 275 at ± 12.5 mg/dL, were individually placed on the PA cell over the resonator cavity at room temperature. A pressure of 6 N/cm$^2$ was applied to the samples, measured by a sensitive pressure sensor (400 FSR, Interlink Electronics, Toronto, ON, Canada). Applying constant pressure throughout the measurements is essential to the results’ compatibility^18^. The 9.25 $\upmu$m and 10.3 $\upmu$m QCLs were operated at 220 mA and 300 mA with an average power of 8 mW and 5 mW, respectively. The fiber-coupled modulated beams of the 10.3 and 9.25 $\upmu$m QCLs were focused on the PA cell cavity in order to generate the acoustic waves. The 10.3 $\upmu$m QCL was run first as a reference light source for the measurements. Each sample was scanned from 10 to 40 khz with a frequency step of 0.15 khz for both lasers. The acoustic waves were amplified inside the PA cavity and collected by a sensitive microphone (SPU0410LR5H-QB). A lock-in amplifier (SR830) processed the collected PA signals with a time constant of 300 ms. The measurements were repeated five times per light source, and the collected acoustic signals were transmitted to the PC through a data acquisition system for further analysis. The experiment was repeated for two days with new samples, following similar procedures.

**Figure 6:**
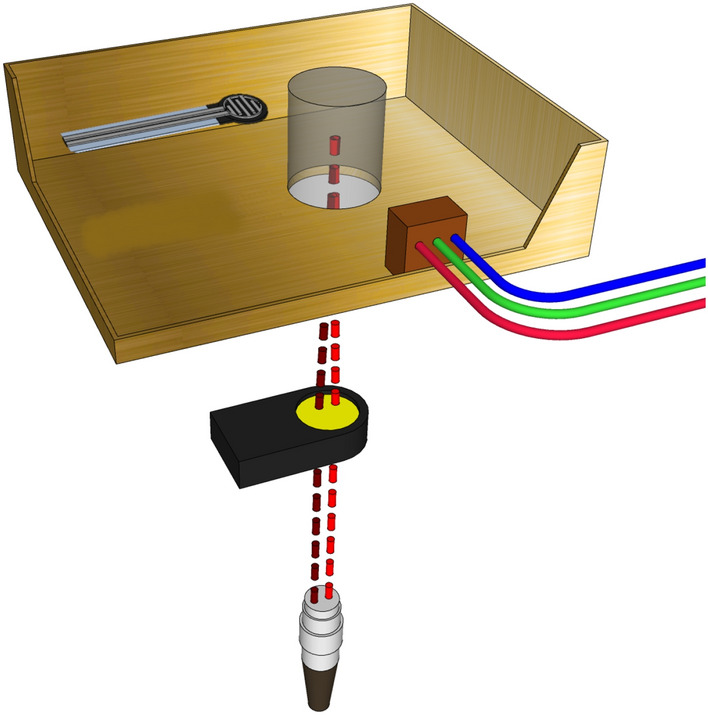
Design of the optical lens.

An IR Plano-Convex lens (ZNPX11, NEWPORT, United States) was installed underneath the PA cavity, as shown in Fig. 6, before conducting the measurements for the second sample set, which contains blood components. The zinc selenide lens is transparent for both lasers and allows them to radiate at the exact spot of the samples, hence generating acoustic waves from an identical source. The last set of measurements was designed to study the effects of the optical lens on the results’ precision. In this measurement set, the glucose samples with blood components were subjected to two days of measurements with and without the lens. When the lens was not installed, the two fiber-coupled lasers radiated parallel to the sample with around a 1 mm overlap between their beam spots on the samples. Table 3 summarizes the process for the three categories of measurements for both sets of samples.

**Table 3:** Summary of the measurement procedures.

Index	Sample no.	Sample contents	Glucose concentration (mg/dL)	9.25 $\upmu$m QCL	10.3 m QCL
Day 1	1st sample	Glucose only	Non-diabetic range (100:25:225) and diabetic range (225:12.5:275)	5 rounds (10:0.15:40 kHz)	5 rounds (10:0.15:40 kHz)
Day 2	2nd sample				
Day 1	1st sample	Glucose, albumin, urea, sodium lactate, and cholesterol	Diabetic and non-diabetic range (100:25:275)	5 rounds (10:0.15:40 kHz)	5 rounds (10:0.15:40 kHz)
Day 2	2nd sample				
Day 1	1st sample with lens	Glucose, albumin, urea, sodium lactate, and cholesterol	Mid range (150:25:250)	5 rounds (10:0.15:40 kHz)	5 rounds (10:0.15:40 kHz)
Day 2	2nd sample without lens				

### Machine learning for glucose detection

An ensemble of bagged decision trees classification model was developed using the unprocessed acoustic signals obtained from glucose detection measurements. The bootstrap aggregating models help improve the prediction accuracy and reduce the over-fitting of the algorithms^28^. The model was trained with 72,360 datasets of the acoustic signals obtained in the measurements for both sets of phantoms. The development of ML models is assisted by producing a large number of datasets for training from both QCLs. In ML, each row represents a dataset, while each column represents a feature. In this work, the data from each sampling frequency was assigned to one column to create a coherent feature with a given glucose class label. For example, the data obtained at 20 khz was designated into one column for all samples versus the class label. The presence of the blood components was assigned into a column in the data arrangement with a binary weight. As a result, the total number of features used in building the model was 403. The data arrangement for the classifier training purposes is shown in Table 4. The model was trained with all frequency features and without pre-processing the data to investigate the robustness of the acoustic signals obtained in the measurements. The number of learners for the model was tuned over the training to maximize the prediction accuracy. The maximum number of splits for the decision tree technique was optimized during the training. The model was evaluated using the k-fold cross-validation of 10-fold. Implementing machine learning in the MIR and PA spectroscopy may assist the development of in vivo measurements, where various stochastic parameters can influence acoustic signals such as human skin differences. Besides, machine learning can effectively substitute the calibration process in order to build a general prediction model for noninvasive glucose detection.

**Table 4:** Dataset arrangement of the glucose acoustic spectrum for ML training purposes.

Sample contents	Sample no.	9.25 $\upmu$m QCL	10.3 $\upmu$m QCL	Presence of b.c.	Class label
Samples with glucose only	First sample	Round 1	Round 1	0	100
		. .	. .	. .	. .
		Round 5	Round 5	0	100
	.	.	.	.	.
	.	.	.	.	.
	Last sample	Round 1	Round 1	0	275
		. .	. .	. .	. .
		Round 5	Round 5	0	275
Samples with other blood components (b.c.)	First sample	Round 1	Round 1	1	100
		. .	. .	. .	. .
		Round 5	Round 5	1	100
	.	.	.	.	.
	.	.	.	.	.
	Last sample	Round 1	Round 1	1	275
		. .	. .	. .	. .
		Round 5	Round 5	1	275

## Supplementary Information

Supplementary Information.

## Data Availability

The datasets used and/or analyzed during the current study are available from the corresponding author upon reasonable request.
