# Author Correction: Dual quantum cascade lasers for noninvasive glucose detection using photoacoustic spectroscopy

**DOI:** 10.1038/s41598-025-91975-0

**Published:** 2025-03-10

**Authors:** Abdulrahman Aloraynan, Shazzad Rassel, Md. Rejvi Kaysir, Dayan Ban

**Affiliations:** 1 Department of Electrical Engineering, Umm Al-Qura University, Makkah, Saudi Arabia; 2 Department of Electrical and Computer Engineering, University of Waterloo, Waterloo, ON N2L 3G1 Canada; 3 Waterloo Institute for Nanotechnology, University of Waterloo, Waterloo, ON N2L 3G1 Canada

Correction to: *Scientific Reports* 10.1038/s41598-023-34912-3, published online 16 May 2023

In the original version of this Article, Affiliation 1, 2 and 3 were not listed in the correct order for author Abdulrahman Aloraynan. The correctly numbered affiliations are listed below:

Affiliation 1:

Department of Electrical Engineering, Umm Al-Qura University, Makkah, Saudi Arabia.

Affiliation 2:

Department of Electrical and Computer Engineering, University of Waterloo, Waterloo, ON, N2L 3G1, Canada.

Affiliation 3:

Waterloo Institute for Nanotechnology, University of Waterloo, Waterloo, ON, N2L 3G1, Canada.

The original Article has been corrected.

